# TAS2R38 bitter taste receptor and attainment of exceptional longevity

**DOI:** 10.1038/s41598-019-54604-1

**Published:** 2019-12-02

**Authors:** Melania Melis, Alessandra Errigo, Roberto Crnjar, Giovanni Mario Pes, Iole Tomassini Barbarossa

**Affiliations:** 1 Department of Biomedical Sciences, University of Cagliari, Monserrato, CA 09042 Italy; 2 Department of Biomedical Sciences, University of Sassari, Sassari, SS 07100 Italy; 3 Department of Medical, Surgical and Experimental Sciences, University of Sassari, Sassari, SS 07100 Italy; 4 Sardinia Longevity Blue Zone Observatory, Ogliastra, Italy

**Keywords:** Ageing, Taste receptors

## Abstract

Bitter taste receptors play crucial roles in detecting bitter compounds not only in the oral cavity, but also in extraoral tissues where they are involved in a variety of non‒tasting physiological processes. On the other hand, disorders or modifications in the sensitivity or expression of these extraoral receptors can affect physiological functions. Here we evaluated the role of the bitter receptor TAS2R38 in attainment of longevity, since it has been widely associated with individual differences in taste perception, food preferences, diet, nutrition, immune responses and pathophysiological mechanisms. Differences in genotype distribution and haplotype frequency at the *TAS2R38* gene between a cohort of centenarian and near-centenarian subjects and two control cohorts were determined. Results show in the centenarian cohort an increased frequency of subjects carrying the homozygous genotype for the functional variant of *TAS2R38* (PAV/PAV) and a decreased frequency of those having homozygous genotype for the non-functional form (AVI/AVI), as compared to those determined in the two control cohorts. In conclusion, our data providing evidence of an association between genetic variants of *TAS2R38* gene and human longevity, suggest that TAS2R38 bitter receptor can be involved in the molecular physiological mechanisms implied in the biological process of aging.

## Introduction

The sense of taste is defined as a sensory system in which taste receptor cells are capable of detecting chemical molecules and provide valuable information about the nature and quality of food, but also about health-related issues^1,2^. In humans, taste receptors were originally identified and named based on their role in the taste cells of the tongue where they can discriminate five basic qualities: sweet, sour, salt, umami and bitter^3^. However, recent studies have shown that taste receptors are also expressed in a numerous extra-oral tissues throughout the body, including the airway and gastrointestinal tract, pancreas, liver, kidneys, testes, bladder and brain where they participate in a variety of physiological processes^4–10^. Although the role of taste receptors in the extra-oral tissues has been partially elucidated, recent studies have shown that they sense chemical molecules by means of transduction mechanisms and chemosensory signalling pathways like those occurring in the taste cells of the tongue^5,8^. Disorders or modifications in the sensitivity or expression of these extra-oral receptors and signalling pathways can affect physiological functions^5^.

Specifically, bitter taste receptors (T2Rs) that belong to the G-protein coupled receptors superfamily, detect many bitter chemicals with different chemical structures and plant-based compounds^11^. Humans possess approximately 25 bitter receptors (TAS2R)^12^. Traditionally, it has been assumed that they initiate bitter taste perception in the oral cavity which serves as a central warning signal against the ingestion of potential toxins. However, growing evidence indicates that T2Rs are widely expressed throughout the body where they mediate diverse non-tasting functions and that their genetic variants are associated with different human disorders^9^.

Among T2Rs, TAS2R38 has been widely studied because it mediates the bitter taste of thiourea compounds, such as phenylthiocarbamide (PTC) and 6-*n*-propylthiouracil (PROP), which has been reported as an oral marker for individual differences in taste perception, general food preferences and dietary behaviour, with consequent links to body mass composition and other non-tasting physiological mechanisms^1,2,9,13–17^. Several results show that perception of to the bitter taste PTC/PROP is associated with perception of other taste stimuli^13,18–25^, food preferences and choices^13,26–29^. Peculiarly, PROP super-taster individuals, compared to PROP non-tasters, seem to show a greater sensitivity and a lower liking and intake for high-fat/high-energy foods^1,20,30^, and a reduced intake of vegetables and fruits^13,16,17,26,28,31,32^. However, other studies did not confirm these associations^33–40^ suggesting that other factors may contribute to dietary predisposition and eating behaviour^2^. PROP perception has also been associated to health markers including: body mass index^28,41^, metabolic changes which impact on body mass composition^42,43^, antioxidant status^44^, colonic neoplasm risk^45,46^, smoking behaviors^47^, consumption of alcoholic beverages^20^, predisposition to respiratory infections^48^ and even neurodegenerative diseases^49–53^. In addition, TAS2R38 has been shown to detect bacterial quorum-sensing molecules and to regulate nitric oxide-dependent innate immune responses of the human respiratory tract^48^.

The gene codifying for TAS2R38 receptor is characterized by three non-synonymous coding single nucleotide polymorphisms (SNPs) which result in two common variants of the TAS2R38 protein: the functional form containing proline, alanine and valine (henceforth the associated genotype is named as PAV) and the non-functional variant containing alanine, valine and isoleucine, (with the genotype named as AVI)^20,54^. TAS2R38 SNPs dictate individual differences in PTC/PROP tasting^54–56^, food linking patterns^1,57^ and also in TAS2R38‒mediated pathophysiology^9^, such as susceptibility, severity, and prognosis of upper respiratory infection, chronic rhinosinusitis and biofilm formation in chronic rhinosinusitis patients^48,58–64^, development of colorectal cancer^45,65^, taste disorders^66^ and neurodegenerative diseases^49^.

Given the implications of TAS2R38 bitter receptor in taste perception, food preferences, diet and nutrition^2^ (which can affect human development and subsequently longevity^67–70^), and those in an efficient immune response^48^ and disease aetiology^9,45,49,66^ (which modulate the physiological mechanisms involved in the biological process of aging^71,72^), it is likely that TAS2R38 and its variants can play an important role in the attainment of longevity.

**Figure 1:**
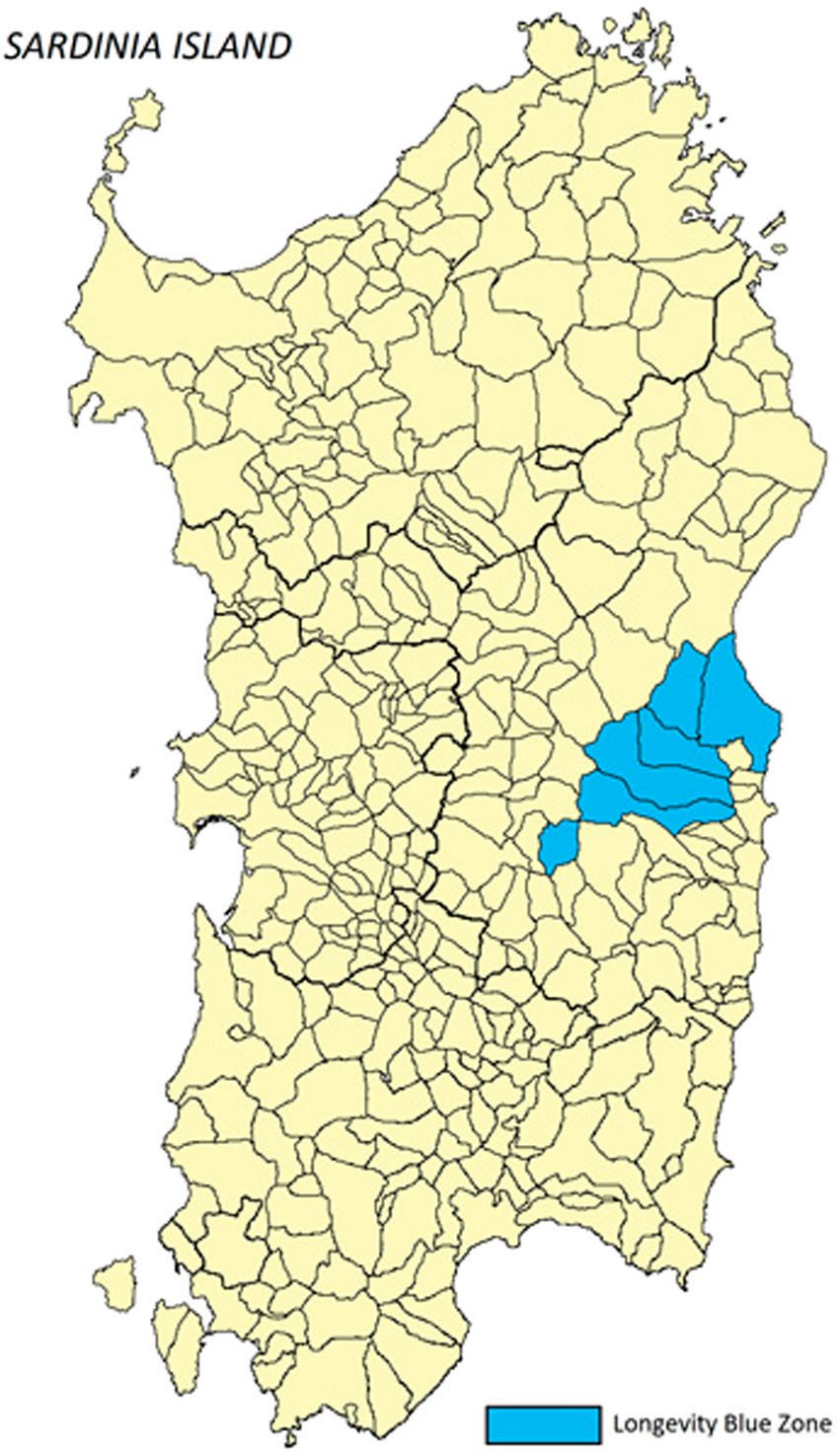
Geographical location of the Longevity Blue Zone villages.

In this study we analysed the genotype distribution and allele frequency of the *TAS2R38* gene in a cohort of centenarian subjects recruited in a genetically isolated area of the central-eastern Sardinia island (called the Longevity Blue Zone, LBZ). This area, which includes six mountainous villages of Ogliastra and Barbagia, has a total population of nearly 12,000 inhabitants on a land area of 888 km^2^ (Fig. 1)^73^. This population has remained isolated for many centuries, which made its genetic make‒up one of the most homogeneous in Europe^74^, and its sociocultural and anthropological characteristics well preserved throughout history^73,75^. LBZ represents an interesting case study because it shows a value of the Extreme Longevity Index (ELI)^76^ computed for generations born between 1880 and 1900 that is more than twice as high as that of the whole Sardinia. For this reason, data from this population were compared with data from ancestrally‒diverse cohorts recruited in another area of Sardinia (area of Cagliari, in the south of Sardinia).

The association between longevity and taste genetics has been already investigated^77,78^. Campa and co-authors^77^ analysed, in a population from Calabria (Italy), the association with longevity of the common genetic variants of three bitter taste receptor genes that are involved in food preferences, food absorption processing and metabolism. They compared the results from centenarian subjects with those from non-elderly controls with a wide age range (20–84 years). Results showed that the frequency of subjects who carried the genotype homozygote AA for the polymorphism, *rs978739*, in *TAS1R16* gene increases gradually from 35% in subjects aged 20 up to 55% in centenarians. However, another study did not confirm this association, by comparing results from centenarian with those from young controls (age range 18–45 years), in a population from another area of South Italy (Cilento) which could be subjected to different demographic pressures^78^. Here, we decided to compare results from centenarian subjects of the LBZ with those of two control cohorts south of Sardinia differentiated based on their age, one of young adult subjects (age ranging from 18 to 35 years) and a second of middle-aged adults and older adults (age ranging from 36 to 85 years).

## Materials and Methods

### Subjects

Three hundred seventy-three subjects were included in the study. They were divided in three groups based on their age and area of Sardinia island (Italy) where they were recruited: the Longevity Blue Zone cohort (LBZ) (*n* = 94) (age ranging from 90 to 105 years) included subjects recruited in the central‒eastern area (Ogliastra/Barbagia) of Sardinia; the Cagliari young cohort (CY) (*n* = 181) (age ranging from 18 to 35 years) included subjects recruited in the area of the city of Cagliari (Sardinia); the Cagliari cohort including middle-aged adults and elder adults (CMAE) (*n* = 98) (age ranging from 36 to 85 years) with subjects recruited in the same area of CY cohort. The demographic features of the three cohorts are summarized in Table 1. All subjects were recruited through public advertisements. Specifically, in the case of LBZ cohort, subjects aged 90 years or older were considered eligible participants for this study and were recruited through the Longevity Blue Zone Observatory ‒ a research center which is systematically collecting demographic and individual data of people ≥65 years old from this area^75^. After excluding residents born outside the LBZ, a blood sample of each eligible subjects was obtained during a home interview. All subjects were informed concerning the procedure and the purpose of the study. All participants provided a signed informed consent form. The study was performed according to the guidelines of the Declaration of Helsinki of 1975 (revised in 1983), and the procedures involving human participants were approved by the ethical committee of the University of Sassari.

**Table 1:** Demographic features of the centenarian cohort and two control cohorts.

	Subjects			Age range (y)
	Total (*n*)	Males (*n*)	Females (*n*)	
*LBZ*	94	42	52	90–105
*CY*	181	57	124	18–35
*CMAE*	98	43	55	36–85

LBZ, Longevity Blue Zone cohort; CY, Cagliari young subjects’ cohort; CMAE, Cagliari cohort including middle-aged adults and elder adults.

### Molecular analyses

DNA was extracted from blood samples by using the QIAamp® DNA Mini Kit (QIAGEN Hilden, Germany) according to the manufacturer’s instructions. Its concentration was assessed by measurements at an optical density of 260 nm with an Agilent Cary 60 UV-Vis Spectrophotometer (Agilent, Palo Alto, CA). All subjects were genotyped for 3 SNPs at base pairs (bp) 145 (C/G), 785 (C/T), and 886 (G/A) of *TAS2R38* and for SNP, *rs1761667* (G/A), by using TaqMan SNP Genotyping Assay (C_8876467_10 assay for the *rs713598*; C_9506827_10 assay for the *rs1726866* and C_9506826_10 assay for the *rs10246939*) according to the manufacturer’s specifications (Applied Biosystems by Life Technologies Milano Italia, Europe BV). Replicates and positive and negative controls were included in all reactions.

### Statistical analyses

Genotype distribution and haplotype frequencies at the *TAS2R38* gene of the three cohorts were compared using Fisher’s method (Genepop software version 4.2; http://genepop.curtin.edu.au/genepop_op3.html)^79^. *P* values < 0.05 were considered significant.

## Results

Molecular analysis of the *TAS2R38* polymorphisms identified in the LBZ cohort (*n* = 94) 32 subjects who were PAV homozygous, 38 heterozygous, 17 AVI homozygous and 7 carried rare haplotypes (3 had AAV/AVI genotype, 2 PAV/AAV, 1 AAV/AAV and 1 AAI/AVI), in the CY cohort (*n* = 181) 41 subjects were PAV homozygous, 85 heterozygous, 48 AVI homozygous and 7 carried rare haplotypes (1 had AAV/AVI genotype, 2 PAV/AAV, 2 PVI/AVI, 1 PAV/AAI and 1 AAI/AVI) and in the CMAE cohort (*n* = 98) 18 subjects were PAV homozygous, 43 heterozygous, 35 AVI homozygous and 2 carried rare haplotypes (1 had PAV/AAV genotype and 1 AAI/AVI) (Supplementary information).

**Figure 2:**
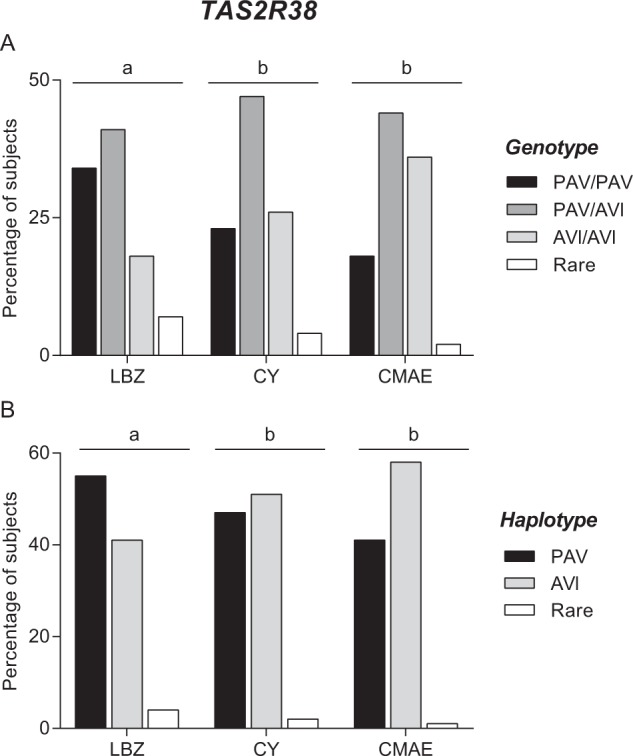
Genotype distribution (**A**) and haplotype frequencies (**B**) of polymorphisms of *TAS2R38* gene in the Longevity Blue Zone cohort (LBZ) (*n* = 94), Cagliari young subjects’ cohort (CY) (*n* = 181) and the Cagliari cohort including middle-aged adults and elder adults (CMAE) (*n* = 98). Different letters indicated significant difference (*Χ*^*2*^ > 6.38; *P* ≤ 0.041; Fisher’s test).

The three cohorts differed statistically based on their genotype distribution (*Χ*^*2*^ = 8.855; *P* = 0.0119; Fisher’s test) and haplotype frequencies (*Χ*^*2*^ = 11.31; *P* = 0.0035; Fisher’s test) (Fig. 2). Pairwise comparisons showed that the LBZ cohort differed from the other two cohorts (genotype: *Χ*^*2*^ > 6.381; *P* ≤ 0.041; Fisher’s test and haplotype: *Χ*^*2*^ > 6.96; *P* ≤ 0.030; Fisher’s test), which did not differ from each other (genotype: *Χ*^*2*^ = 2.667; *P* = 0.26; haplotype: *Χ*^*2*^ = 2.862; *P* = 0.239; Fisher’s test). The LBZ cohort was characterized by a high frequency of the diplotype PAV/PAV (34.04%) and haplotype PAV (55.32%) and a low frequency of diplotype AVI/AVI (18.08%) and haplotype AVI (40.42%), whereas the CY and CMAE cohorts were characterized by a lower frequency of diplotype PAV/PAV (CY: 22.65%; CMAE: 18.36%) and haplotype PAV (CY: 46.96%; CMAE: 40.81%) and a higher frequency of diplotype AVI/AVI (CY: 26.52%; CMAE: 35.71%) and haplotype AVI (CY: 51.10%; CMAE: 58.16%).

Fisher’s test showed no differences of the genotype distribution and haplotype frequencies between males and females in the three cohorts (LBZ: genotype: *Χ*^*2*^ = 1.0798; *P* = 0.583; haplotype: *Χ*^*2*^ = 1.435; *P* = 0.488; CY: genotype: *Χ*^*2*^ = 0.822; *P* = 0.640 haplotype: *Χ*^*2*^ = 0.538; *P* = 0.764; CMAE: genotype: *Χ*^*2*^ = 1.095; *P* = 0.578; haplotype: *Χ*^*2*^ = 2.733; *P* = 0.255) (Table 2).

**Table 2:** Genotype distribution and haplotype frequencies of polymorphisms of *TAS2R38* gene in males and females of the centenarian cohort and two control cohorts.

	Males		Females		*p*-value^a^
	(*n*)	%	(*n*)	%	
***LBZ***					
*Genotype*					
PAV/PAV	16	38.10	16	30.77	0.583
PAV/AVI	17	40.48	21	40.38	
AVI/AVI	6	14.28	11	21.16	
rare	3	7.14	4	7.69	
*Haplotype*					
PAV	50	59.52	54	51.92	0.488
AVI	30	35.72	46	44.23	
rare	4	4.76	4	3.85	
***CY***					
*Genotype*					
PAV/PAV	14	24.56	27	21.77	0.640
PAV/AVI	25	43.87	60	48.39	
AVI/AVI	17	29.82	31	25	
rare	1	1.75	6	4.84	
*Haplotype*					
PAV	54	47.37	116	46.77	0.764
AVI	59	51.75	126	50.81	
rare	1	0.878	6	2.42	
***CMAE***					
*Genotype*					
PAV/PAV	5	11.63	13	23.63	0.578
PAV/AVI	19	44.19	24	43.64	
AVI/AVI	18	41.86	17	30.91	
rare	1	2.32	1	1.82	
*Haplotype*					
PAV	30	34.89	50	45.46	0.255
AVI	55	63.95	59	53.63	
rare	1	1.16	1	0.91	

^a^p-value derived from Fisher’s method. LBZ, Longevity Blue Zone cohort, *n* = 94 (males: *n* = 42, females: *n* = 52); CY, Cagliari young subjects’ cohort, *n* = 181 (males: *n* = 57, females: *n* = 124); CMAE, Cagliari cohort including middle-aged adults and elder adults, *n* = 98 (males: *n* = 43, females: *n* = 55).

## Discussion

In the present work we studied the role of the bitter receptor, TAS2R38 and its genetic variants on longevity. Our results show that the genetically homogeneous cohort of subjects ranging in age from 90 to 105 years of the an area, which was recognised as one of the world’s longevity hot spots (Longevity Blue Zone)^80^, differed based on the genotype distribution and haplotype frequencies of *TAS2R38* gene from the two genetically heterogeneous cohorts from the South of Sardinia where the longevity level is distinctly lower. Conversely, no differences were found between these two latter cohorts. Specifically, the centenarian cohort showed an increased frequency of subjects carrying the homozygous genotype for the dominant haplotype (PAV/PAV) (34.04%) and haplotype PAV (55.32%) and a reduced frequency of subjects who had the homozygous genotype for the recessive haplotype (AVI/AVI) (18.08%) and haplotype AVI (40.42%). Otherwise, the frequencies determined in the two cohorts from the South of the island, which had a prevalence of subjects carrying the homozygous genotype AVI/AVI (26.52% and 35.71%) and haplotype AVI (51.10% and 58.16%), were similar to those already reported for the Caucasian population in this area^25,81–85^.

In most populations, females have been reported to live longer than males, with geographical differences in the female/male ratio^86^. However, the population living in the Sardinian LBZ is an exception among the other long‒lived populations since the female/male ratio in the oldest old is close to 1:1^80^. In addition, several studies have shown that PROP phenotype and genotype variations are associated with gender, with females being more responsive than males^14,87^. We did not find an effect of gender on the genotype distribution and haplotype frequencies of *TAS2R38* locus in the three cohorts studied in this work. By considering specifically the centenarian cohort (which includes a balanced number of males and females), this result allowed us to exclude that the increased frequency of subjects carrying the genotype PAV/PAV and haplotype PAV and the reduced frequency of subjects with the genotype AVI/AVI and haplotype AVI, that we found in this cohort, can to be due to gender bias.

It is well known that an improving diet aimed at increasing intake of fruits and vegetables instead of fat-rich foods may control obesity and reduce the risk of several diseases^88,89^. A number of studies on human nutrition have suggested that the *TAS2R38* variants and the related PROP phenotype may influence dietary behaviour and nutritional status^1^. The possible association between PROP responsiveness and perception and intake of fats has been extensively studied, but with controversial results^1,13,16,17,20,26,28,30,33,34,38–40,90^. The widely accepted hypothesis is that PROP non-tasters, compared to PROP super-tasters, show a reduced ability to perceive dietary fat which could lead them to increase the consumption of high-fat foods to compensate the reduced perception^57^. In agreement with this assumption, the high frequency of the tasting homozygous genotype (PAV/PAV) and the low frequency of the non-tasting one (AVI/AVI), that we found in centenarian subjects, suggest that these individuals may have reached an exceptional longevity because of their genetic predisposition to a low-fat diet. On the other hand, the extreme bitterness intensity of PROP super-tasters has been shown to be the primary reason for avoiding bitter-tasting fruits and vegetables^57,91^. Since many bitter-tasting compounds in foods (e.g., flavonoids, phenols, glucosinolates) have benefit effects for health^92,93^, our results in the centenarian cohort seem to be in contrast with the possibility that *TAS2R38* genotype is a genetic factor that favour an adequate intake of fruits and vegetables or other bitter foods recommended for a healthy life. However, only a few studies have investigated the relationship between *TAS2R38* variants and vegetable intake obtaining controversial results^35,38,91,94^. Although, the notion that TAS2R38 might serve to govern food intake is interesting, eating behaviour is a complex phenomenon influenced by a broad range of environmental factors, including social cues, socioeconomic status, culture and education, as well as by individual features, such as gender, body weight, ethnicity and health status^57^. All these factors will be considered in future studies aimed at analysing the role of *TAS2R38* variants in the diet of centenarians.

In addition, it is known that TAS2R38 receptor serves other genotype-dependent roles which are relevant for health, with the PAV form associated with an efficient immune response^9,48,58,60,64,95–97^, a favourable body composition^1,32,42,98^, as well as with physiological processes^59,61–63^. On the contrary, the AVI group is associated with a higher risk to develop many dysfunctions and diseases^45,48,49,58–66^. Therefore, it is not surprising that we find in the centenarian cohort an increased frequency of homozygous subjects for the functional variant of *TAS2R38* (PAV) and above all a decreased frequency of those having homozygous genotype for the non-functional form (AVI).

In conclusion, our findings providing evidence of an association between genetic variants of *TAS2R38* gene and human longevity, highlight the role of the G protein-coupled receptor (GPCR), TAS2R38 in the molecular physiological mechanisms of the bitter perception associated with important factors involved in the biological process of aging, and suggest that individuals who have a pair of functional alleles (PAV/PAV) at *TAS2R38* gene may have a favourable genetic condition for the attainment of exceptional longevity.

## Supplementary information

Table S-1
