# Supplementary material for: TAS2R38 bitter taste receptor and attainment of exceptional longevity
Source: Sci Rep. 2019 Dec 2;9:18047. doi: 10.1038/s41598-019-54604-1 (PMC6889489; doi:10.1038/s41598-019-54604-1)
Supplement: Supplementary file 1 — Table S-1 [file 41598_2019_54604_MOESM1_ESM.docx]

**Supplementary Information for:**

**TAS2R38 bitter taste receptor and attainment of exceptional longevity**

Melania Melis^1*^, Alessandra Errigo^2^, Roberto Crnjar^1^, Giovanni Mario Pes^3,4^, Iole Tomassini Barbarossa^1^

^1^ Department of Biomedical Sciences, University of Cagliari, Monserrato, CA 09042, Italy.

^2^ Department of Biomedical Sciences, University of Sassari, SS 07100, Italy.

^3^Department of Medical, Surgical and Experimental Sciences, University of Sassari, SS 07100, Italy.

^4^Sardinia Longevity Blue Zone Observatory, Ogliastra, Italy

Table S-1. Genotype distribution and haplotype frequencies of polymorphisms of *TAS2R38* gene in the Longevity Blue Zone cohort (LBZ), Cagliari young subjects’ cohort (CY) and the Cagliari cohort including middle-aged adults and elder adults (CMAE).

| **TAS2R38** | |  |  |  |  |  |  |  |  |
| --- | --- | --- | --- | --- | --- | --- | --- | --- | --- |
| **LBZ** | **Genotype** |  |  |  |  | **Haplotype** | |  |  |
|  |  | *n* | % |  |  |  | *n* | % |  |
|  | **PAV/PAV** | **32** | **34,043** |  |  | **PAV** | 104 | 55,319 |  |
|  | **PAV/AVI** | **38** | **40,426** |  |  | **AVI** | 76 | 40,426 |  |
|  | **AVI/AVI** | **17** | **18,085** |  |  | **Rare** | 8 | 4,255 |  |
|  | PAV/AAV | 2 | 2,128 |  |  |  | 188 |  |  |
|  | AAV/AAV | 1 | 1,064 |  |  |  |  |  |  |
|  | AAV/AVI | 3 | 3,191 |  |  |  |  |  |  |
|  | AAI/AVI | 1 | 1,064 |  |  |  |  |  |  |
|  | **Rare** | 7 | 7,447 |  |  |  |  |  |  |
|  |  | 94 |  |  |  |  |  |  |  |
|  |  |  |  |  |  |  |  |  |  |
|  |  |  |  |  |  |  |  |  |  |
| **CY** | **Genotype** |  |  |  |  | **Haplotype** | |  |  |
|  |  | *n* | % |  |  |  | *n* | % |  |
|  | **PAV/PAV** | **41** | **22,652** |  |  | **PAV** | 170 | 46,961 |  |
|  | **PAV/AVI** | **85** | **46,961** |  |  | **AVI** | 185 | 51,105 |  |
|  | **AVI/AVI** | **48** | **26,519** |  |  | **Rare** | 7 | 1,934 |  |
|  | AAI/AVI | 1 | 0,552 |  |  |  | 362 |  |  |
|  | PAV/AAV | 2 | 1,105 |  |  |  |  |  |  |
|  | PAV/AAI | 1 | 0,552 |  |  |  |  |  |  |
|  | PVI/AVI | 2 | 1,105 |  |  |  |  |  |  |
|  | AAV/AVI | 1 | 0,552 | 3,8674 |  |  |  |  |  |
|  | **Rare** | 7 |  |  |  |  |  |  |  |
|  |  | 181 |  |  |  |  |  |  |  |
|  |  |  |  |  |  |  |  |  |  |
| **CMAE** | **Genotype** |  |  |  |  | **Haplotype** | |  |  |
|  |  | *n* | % |  |  |  | *n* | % |  |
|  | **PAV/PAV** | **18** | **18,367** |  |  | **PAV** | 80 | 40,816 |  |
|  | **PAV/AVI** | **43** | **43,878** |  |  | **AVI** | 114 | 58,163 |  |
|  | **AVI/AVI** | **35** | **35,714** |  |  | **Rare** | 2 | 1,064 |  |
|  | AAI/AVI | 1 | 1,020 |  |  |  | 196 |  |  |
|  | PAV/AAV | 1 | 1,020 | 2,041 |  |  |  |  |  |
|  | **Rare** | 2 |  |  |  |  |  |  |  |
|  |  | 98 |  |  |  |  |  |  |  |
